# Is Serum Lactate Necessary in Patients with Normal Anion Gap and Serum Bicarbonate?

**DOI:** 10.5811/westjem.2015.2.23906

**Published:** 2015-04-02

**Authors:** Daniel Aronovich, Maykel Trotter, Cynthia Rivera, Michael Dalley, David Farcy, Michel Betancourt, Lydia Howard, Sharon Licciardi, Luigi Cubeddu, Robert Goldszer

**Affiliations:** * Mount Sinai Medical Center, Department of Emergency Medicine, Miami Beach, Florida; † Mount Sinai Medical Center, Department of Internal Medicine, Miami Beach, Florida; ‡ Mount Sinai Medical Center, Department of Pathology, Miami Beach, Florida; § Nova Southeastern University, Health Professions Division, Fort Lauderdale, Florida

## Abstract

**Introduction:**

There has been an increase in patients having serum lactate drawn in emergency situations. The objective of this study was to determine whether or not it was necessary to obtain a lactate level in patients with a normal serum bicarbonate level and anion gap.

**Methods:**

This is a retrospective chart review evaluation of 304 patients who had serum lactate and electrolytes measured in an emergency setting in one academic medical center.

**Results:**

In 66 patients who had elevated serum lactate (>2.2mmol/L), 45 (68%) patients had normal serum bicarbonate (SB) (greater than 21 mmol/L). Normal anion gap (AG) (normal range <16 mEq/l) was found in 51 of the 66 patients (77%).

**Conclusion:**

We found that among patients with elevated serum lactate, 77% had a normal anion gap and 68% had normal serum bicarbonate. We conclude serum lactate should be drawn based on clinical suspicion of anaerobic tissue metabolism independent of serum bicarbonate or anion gap values.

## INTRODUCTION

A variety of laboratory parameters can help identify patients with severely compromised or strained metabolism. Among these are the anion gap (AG), serum bicarbonate (SB), pH, and serum lactate (SL) levels. There are two possible strategies for the diagnostic detection of lactic acidosis. The first strategy is to order a lactate level upon any clinical suspicion of acidosis. The second strategy is to order routine chemistry and then if there is abnormality order follow up tests such as a serum lactate.

While the presence or absence of an AG has classically been used as a screening tool for lactic acidosis, there are some potential problems with this stepwise strategy.^1^ Firstly, it has been recently suggestedthat the upper limit of a “normal” AG should be lowered to six because of a technological change in the process that measures electrolyte concentrations.^1^ This is currently not accepted. Using a lower AG threshold would increase the number of subsequently ordered lactates. Secondly, lactic acidosis is a marker of life-threatening illness, and any delay between recognizing an increased AG level and then ordering and confirming a lactate level may add unnecessary risk to the patient. In one retrospective cohort study, Adams et al. evaluated all emergency department (ED) patients seen over a seven-month period in whom a lactate level was measured for any reason. The authors considered an AG >12 abnormal and conducted sensitivity analyses of the AG for detecting the presence of a lactate >2.5mmol/L. The AG was 52.8% sensitive, 81.0% specific, and with a negative predictive value of 89.7% for lactic acidosis.^1^ Critically ill patients have impaired acid-base regulation and are thought to generate more unmeasured cations, such as magnesium and calcium, thereby affecting the AG. Furthermore, hypo-albuminemia affects the AG and is also prevalent in the ED population.^1^,^2^ From these prior studies, it appears that the AG cannot be considered a surrogate for lactate testing.

## METHODS

We attempted to answer the question, is it necessary to draw a serum lactate if a patient has normal anion gap and normal serum bicarbonate? Our hypothesis was that it may not be necessary to draw serum lactate if a patient had no electrolyte suggestion of metabolic acidosis. Perhaps we could save time and money and draw less lab tests for patient evaluation. This was a retrospective chart review study of patients who received the index test (venous lactate level) in the ED or as an inpatient for any clinical reason. The a priori dependent variables used in the analysis were age, gender, date of test, time of lactate drawn, serum lactate level, time of electrolytes drawn, bicarbonate level, anion gap, and creatinine. Data extraction was performed by all co-investigators who were aware of the study hypothesis (non-blinded) and who were all educated on data extraction and input on a prepared electronic template. We did not assess inter-rater reliability. Only patients with complete data were included. We performed data acquisition using a computer-generated search for consecutive patients in whom a serum lactate was drawn.^4^,^5^ Serum lactate was drawn at the physician’s order based on suspicion of shock or abdomen disease that could lead to shock. Our institutional review board approved the study.

The setting was a large, urban teaching hospital with over 700 beds. Participants were all patients who had a serum lactate level obtained in the ED or as an inpatient. We performed chart review for analysis of 304 consecutive patients who had a serum lactate level starting in 2010. A total of 165 patients had their tests drawn in the ED, and 139 had their tests drawn as inpatients. Two hundred one patients had electrolytes and serum lactate drawn simultaneously. The median for the time difference between SL and electrolytes drawn is zero (25th percentile for median = 0 and 75th percentile for median = 2 hours). We used the normal ranges as now used in our hospital laboratory. Normal serum bicarbonate is 21–32mmol/L; normal anion gap is 5–15. We report only patients who had serum bicarbonate (SB) < 21mmol/L and anion gap (AG) greater than 16mEq/L, and normal serum lactate is less than 2mmol/L. Results were expressed as either mean values ± standard error of the mean, median (25th percentile to 75^th^ percentile) as absolute numbers or as percentages. We assessed statistical difference between means by Student’s t test (unpaired, two tails). We tested ratios or percentages by chi square test. Differences were considered significant at values of p <0.05.

## RESULTS

Demographics and lab values of the 304 patients analyzed are found in Table 1. Serum lactate, bicarbonate and anion gap levels averaged 1.99mmol/L, 26.1mmol/L, and 14.5mEq/L, respectively. Patients with serum lactate levels equal to or greater than 2.2mmol/L (n=66) had statistically significant lower bicarbonate and anion gap than those with normal serum lactate (Table 1). Significant negative associations were found between serum lactate and serum bicarbonate (p< 0.001). Only 35 (11.5%) of all 304 patients had an anion gap greater than 16.

In the 66 patients (Table 2) who had elevated serum lactate (>2.2mmol/L), 45 (68.1%) had serum bicarbonate greater than 21 mmol/L (normal range 21–32mmol/L). Anion gap less than 16 (normal range 5–15mEq/L) was found in 51 of the 66 patients (77.2%). In the 22 patients with SL greater than 4mmol/L there were 10/22 (45.5%) with SB greater than 21 and 12/22 (54.6%) with AG less than 16mEq/L. Our findings indicate that a serum lactate may be elevated despite normal serum bicarbonate and anion gap values.

## DISCUSSION

Serum lactate is now used commonly in hospitals to assist with diagnosis and management of patients presenting with signs and symptoms of sepsis and/or shock. Prior studies have shown elevated levels are consistent with metabolic changes of decreased tissue perfusion.^3^ Other commonly done tests such as anion gap and serum bicarbonate can be abnormal in patients with similar pathophysiology. Most patients arriving in EDs will have emergency measurement of electrolytes. In this study we asked if it is necessary to draw a serum lactate in patients who do not show signs of metabolic acidosis by elevation of anion gap and decreased serum bicarbonate. In our series of 304 consecutive patients who had SL measured we found 86.6% with SB greater than 21mmol/L and 88.5% with AG less than 16mEq/L. Thus, we conclude serum lactate should be drawn based on clinical suspicion of anaerobic tissue metabolism independent of serum bicarbonate or anion gap values.

There are several possible reasons why SL could be high while anion gap and serum bicarbonate remain normal. One possibility is that cellular metabolism could be changing when the blood is being drawn. Another possibility is that serum lactate could be more sensitive than anion gap or serum bicarbonate. Or perhaps what we are currently describing as normal serum lactate, <2mmol/L, is too low and normal should readjusted to 3mmol/L.

We believe it is important to critically evaluate increasingly common laboratory testing to provide high quality, evidence-based, and high-value care.

## LIMITATIONS

Our study has several limitations. As a retrospective chart review, we may have missed appropriate patients in the electronic medical record. In 201 of the 304 patients lab tests were done simultaneously. It is possible with resuscitation that there were changes in lab values. Patients were identified by having had a serum lactate result; thus, the clinicians had an index of suspicion for altered cellular metabolism.

## CONCLUSION

Our findings revealed a high percentage of patients with abnormal serum lactate and yet normal serum bicarbonate and anion gap. Seventy-seven percent of patients with elevated lactate have normal AG and 68% have normal bicarbonate. Our study indicates that lactate levels can be elevated independent of anion gap and serum bicarbonate levels, and thus should be drawn based on clinical suspicion of cellular hypoxemia.

## Figures and Tables

**Table 1:** Patient demographics and serum electrolyte levels.

Serum lactate levels (mmol/L)	Age (years)	Serum lactate (mmol/L)	Serum bicarbonate (mmol/L)	Anion gap (mEq/L)
All patients (n=304)	68.3 ± 1.1	1.99 ± 0.13	26.1 ± 0.30	14.5 ± 0.30
SL < 2.2 mmol/L(n=238)	67.3 ± 1.2	1.21 ± 0.03	27.1 ± 0.3	13.5 ± 1.5
SL ≥ 2.2 mmol/L (n=66)	72.2 ± 2.2	4.78 ± 0.5	23.1 ± 0.8	18.2 ± 3.3
	p=0.06		p=0.0001	p=0.043

*SL,* serum lactate

Shown are mean values ± SEM for n observations.

**Table 2:** Distribution of patients with low serum bicarbonate and high anion gap levels according to serum lactate levels.

Serum lactate levels (mmol/L)	Serum bicarbonate <21mmol/L n (%)	AG levels >16mEq/L n (%)
All subjects (n=304)	41 (13.4%)	35 (11.5%)
SL <2.2mmol/L (n=238)	20 (8.4%)	20 (8.5 %)
SL ≥2.2mmol/L (n=66)	21 (31.8%)^a^	15 (22.8%)^b^
SL ≥4mmol/L (n=22)	12 (54.5%)	10 (45.4%)

*SL*, serum lactate; *AG*, anion gap

a Statistically significant differences between patients with serum lactate levels lower than 2.2 and patients with serum lactate equal or higher than 2.2mmol/L, all patients had serum bicarbonate levels lower than 21mmol/L (Chi square=24.7; p=0.00006).

b Statistically significant differences between patients with serum lactate levels lower than 2.2 and patients with serum lactate equal or higher than 2.2mmol/L, all patients had AG levels greater than 16mEq/L (Chi square=14.6; p=0.00013).
